# Occupational exposure to asbestos and mortality among asbestos removal workers: a Poisson regression analysis

**DOI:** 10.1038/sj.bjc.6604564

**Published:** 2008-08-19

**Authors:** G Frost, A-H Harding, A Darnton, D McElvenny, D Morgan

**Affiliations:** 1 Health & Safety Laboratory, Harpur Hill, Buxton, Derbyshire SK17 9JN, UK; 2 Health & Safety Executive, Redgrave Court, Bootle, Merseyside L20 7HS, UK

**Keywords:** asbestos, mortality, Great Britain, occupational exposure, asbestos removal

## Abstract

The asbestos industry has shifted from manufacture to stripping/removal work. The aim of this study was to investigate early indications of mortality among removal workers. The study population consisted of 31 302 stripping/removal workers in the Great Britain Asbestos Survey, followed up to December 2005. Relative risks (RR) for causes of death with elevated standardised mortality ratios (SMR) and sufficient deaths were obtained from Poisson regression. Risk factors considered included dust suppression technique, type of respirator used, hours spent stripping, smoking status and exposure length. Deaths were elevated for all causes (SMR 123, 95% CI 119–127, *n*=985), all cancers including lung cancer, mesothelioma, and circulatory disease. There were no significant differences between suppression techniques and respirator types. Spending more than 40 h per week stripping rather than less than 10, increased mortality risk from all causes (RR 1.4, 95% CI 1.2–1.7), circulatory disease and ischaemic heart disease. Elevated mesothelioma risks were observed for those first exposed at young ages or exposed for more than 30 years. This study is a first step in assessing long-term mortality of asbestos removal workers in relation to working practices and asbestos exposure. Further follow-up will allow the impact of recent regulations to be assessed.

Asbestos is a naturally occurring mineral with properties of heat and flame resistance, insulation, flexibility and strength, which made asbestos popular in the early twentieth century, especially in cars, buildings and many domestic products. It was not until the mid-1920s that a link with disease started to be discussed. This led to implementation of the first legislation in Britain in 1931 to reduce occupational exposure of asbestos workers (Bartrip, 2004).

Subsequent regulations, importation bans and the prohibition of asbestos installation changed the nature of the industry. Previously, most asbestos workers were employed in manufacture, but this has now shifted so that those employed in stripping/removal occupations make up the majority (Harding and Wegerdt, 2006).

The key tasks involved in asbestos removal include preparing the work area, removing the substance, bagging the debris, and cleaning up the site area (Williams *et al*, 2007). These workers therefore cause severe disruption to the fibres and come into contact with various forms. Working practices, including removal techniques and personal protective equipment, have been developed to reduce the exposure of workers to asbestos (Ewing and Spain, 1984). It has even been suggested that the stripping/removal industry has become so over-regulated that workers are unlikely to experience elevated exposure to airborne asbestos (Lange *et al*, 2006; Williams *et al*, 2007).

We have investigated the association between mortality and various risk factors associated with asbestos exposure and the stripping/removal industry.

## Materials and methods

The Great Britain Asbestos Survey was established in 1971 to monitor mortality among workers in the asbestos manufacturing industry. Workers were initially invited to participate in the survey with voluntary medical examinations at 2 yearly intervals. Under the Asbestos Licensing Regulations (ALR) 1983 all individuals working with certain kinds of asbestos were required to undergo statutory examinations including pre-employment examinations. At this time substantial numbers of asbestos strippers were recruited into the survey. Our analysis was restricted to the sub-cohort of workers only ever employed as asbestos strippers.

At each medical examination workers completed the survey questionnaire, which included details of the date of first occupational exposure to asbestos and smoking habits. After the introduction of the Control of Asbestos at Work Regulations (CAWR) 1987, the questionnaire was changed to collect more detailed information about asbestos exposures. For removal workers this included information on the type of dust suppression technique used, the kind of respirator used and the weekly hours spent in a stripping enclosure while removal was going on.

Survey participants were flagged for death registrations at the National Health Service Central Register (NHSCR). Data collected at follow-up medical examinations were used to update smoking status and job details. Deaths occurring until December 2005 were included in the analysis. Mesothelioma deaths were coded according to the International Classification of Diseases revision 10 (ICD-10), and so deaths from mesothelioma included in the analysis occurred during 2001 to 2005 only.

### Statistical analysis

Standardised mortality ratios (SMR) were calculated for all workers in the survey only ever employed in asbestos removal work (*n*=52 387). The expected number of deaths was calculated using the 5-year age-, period- and sex-specific mortality rates for England and Wales, and for Scotland. Person-years at risk were calculated from the date of the first medical examination. The SMRs were calculated using OCMAP-PLUS V4.00 (Release 01e) (Marsh *et al*, 2004).

Poisson regression was employed to estimate the relative risks (RR) of mortality among stripping/removal workers. Only those workers who had completed the more recent detailed questionnaire were included in the analysis (*n*=31 302). The dependent variable was the number of deaths, with the person-years at risk as offset variable. Person-years were calculated from the date of first occupational exposure to asbestos as the starting date, and date of death, loss to follow-up or the end of the study period as the ending date. Only causes of death that had significantly elevated SMRs and sufficient deaths (greater than 20) were analysed using Poisson regression.

Relative risks were calculated with adjustment for age (5-year classes, 25–70+ years), calendar period (5-year periods, 1990–2000+) and sex. The covariates of interest were the stripping/removal-specific variables (dust suppression method, respirator type used, and weekly hours spent stripping), smoking status, age at first exposure, length of time in the survey (short- or long-term workers), length of exposure to asbestos and time of first exposure.

Short-term workers were participants who attended only one medical examination, with long-term workers having attended two or more. The dust suppression method was classified into the two techniques of wet and dry removal. There were six categories for respirator type: positive pressure mask, air stream helmet, full-face unpowered mask, half-face mask, minimal and none. Weekly hours spent stripping was summarised in categories (0–9, 10–19, 20–29, 30–39 and 40+ h). Workers who attended more than one examination were allocated to the suppression method and the respirator type they had recorded most often. Categories of ‘both’ suppression techniques and ‘mixed’ respirator use were created to allow for ties to avoid the introduction of any bias by allocating these events to a particular category. Weekly hours spent stripping was taken as the average recorded over all of the worker's examinations.

All variables were entered as a series of indicator variables, the significance of which was tested using the likelihood-ratio test of goodness-of-fit (significance, *P⩽*0.05). Where possible, groupings were combined to eliminate categories with five or less observed cases. All analyses were carried out in Stata 9 (StataCorp, 2005).

## Results

In total, 52 387 asbestos removal workers took part in the survey between 1971 and 2005. Ninety-eight percent of workers were traced for follow-up with the NHSCR. Altogether 31 302 asbestos removal workers, who attended between one and 19 examinations during the study period, were included in the analysis (Table 1). Among the removal workers there were 985 deaths including 384 cancers, 115 lung cancers, and 23 mesotheliomas (Table 2). Statistically significant excesses of deaths from all causes, all cancers, and cancers of the rectum, larynx, lung, peritoneum, pleura and kidney, and mesothelioma were observed (Table 2). There were also significant excesses of deaths from circulatory disease, cerebrovascular disease, respiratory disease and asbestosis (Table 2).

The causes of death with significantly elevated SMRs and sufficient deaths, and therefore included in the Poisson regression analyses, were all causes, all cancers, cancer of the trachea, bronchus and lung, mesothelioma, circulatory disease, ischaemic heart disease, cerebrovascular disease and respiratory disease (Table 2). The characteristics of the asbestos removal workers are described in Table 1. The relative risks obtained using Poisson regression by cause of death and by risk factor are summarised in Table 3.

The majority of workers mainly used the wet removal technique (56%) (Table 1). There were no statistically significant differences in risk between using mainly the wet technique and using the dry method (Table 3).

The positive pressure mask was the main respirator used by the majority of participants (68%) (Table 1). No significant association between risk for any of the diseases of interest and the main respirator used was observed (Table 3).

**Figure 1:**
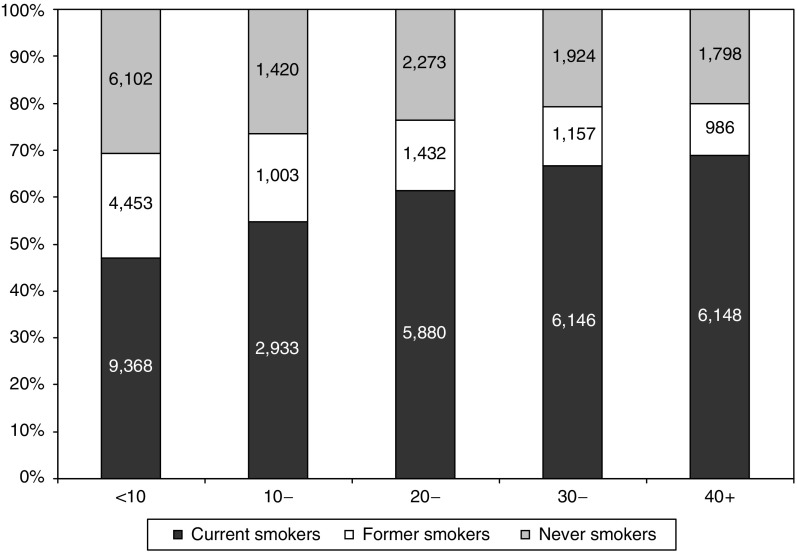
Distribution of examinations by weekly hours spent stripping and smoking status.

The majority of participants (nearly 60%) spent, on average, more than 10 h a week in the stripping enclosure (Table 1). As the number of hours spent in the stripping enclosure increased, the proportion of current smokers also increased (Figure 1). There was a significant increase in risk for workers who spent more than 40 h as compared with less than 10 h a week in the enclosure for all causes, circulatory disease and ischaemic heart disease (all causes: RR 1.4, 95% CI 1.2–1.7; circulatory disease: RR 1.7, 95% CI 1.2–2.4; ischaemic heart disease: RR 1.9, 95% CI 1.2–2.8). Adjustment for smoking status attenuated the results but the observed increasing trend remained (not shown).

The majority of participants were current smokers at their final examination (57%) (Table 1). Mortality from all causes, all cancers, lung cancer, circulatory disease, ischaemic heart disease, and respiratory disease was statistically significantly higher among current smokers than never smokers (Table 3).

The majority of workers (43%) were 20–29 years of age when first exposed to asbestos (Table 1). A reduction in mortality risk for all cancers and for mesothelioma with increasing age of first occupational exposure was observed, and the risk of mortality for all causes was lower for those whose first exposure to asbestos was after age 50 years (RR 0.7, 95% CI 0.6–0.9) (Table 3).

The majority of removal workers (57%) completed just one examination during the study period (Table 1). A statistically significant increase in the risk of mortality from all cancers, lung cancer, and mesothelioma was seen among long-term as compared with short-term workers (all cancers: RR 1.5, 95% CI 1.2–1.9; lung cancer: RR 1.6, 95% CI 1.1–2.4; mesothelioma: RR 4.5, 95% CI 1.3–16.0; Table 3).

The majority of workers (76%) experienced less than 10 years of occupational exposure to asbestos, with only 1% having more than 40 years of exposure (Table 1). An increase in mortality risk for all causes, all cancers and mesothelioma with increasing duration of exposure was observed (Table 3). Mortality risks for all cancers and mesothelioma for those with at least 30 years exposure were statistically higher than those with less than 10 and 30 years exposure respectively (all cancers, 30–39: RR 1.4, 95% CI 1.1–1.9; mesothelioma, 30+: RR 7.3, 95% CI 2.5–21.6; Table 3).

The majority of participants were first occupationally exposed to asbestos post-ALR (83%) (Table 1). The risk of mortality from all cancers and from mesothelioma was significantly lower for those first exposed post-ALR compared with those exposed pre-ALR (Table 3) (all cancers: RR 0.8, 95% CI 0.7–1.0; mesothelioma: RR 0.2, 95% CI 0.04–0.6).

## Discussion

The main strength of the study is that it captured the vast majority of asbestos removal workers covered by the regulations in Great Britain (GB), together with such confounders as smoking status and on the working practices of the participant specific to the asbestos removal industry.

The majority of removal workers included in the analysis (over 80%) were first occupationally exposed to asbestos after the introduction of the ALR (1983). The latency period for asbestos-related diseases is 10–40 or more years (Levin *et al*, 1998; Yarborough, 2006) so any such diseases were beginning to emerge in the survey time frame. However, a longer follow-up period would begin to capture the full extent of asbestos-related disease.

Workers recruited under the 1983 ALR were required to attend pre-employment examinations and so would be unable to answer the removal worker-specific questions. Also, questionnaires completed before 1987 did not include these questions. Altogether, 51% of records had at least one missing response for the detailed stripping/removal variables and 41% of records had missing entries in all of these.

Wetting the area where removal is being carried out controls asbestos fibres and reduces airborne contamination (Paik *et al*, 1983; Sawyer *et al*, 1985; Lange and Thomulka, 2002), so such techniques have become the recommended working practice over dry methods. This study found no evidence that the use of mainly wet suppression techniques reduced the risk of mortality for workers. However, it is possible that this result is due to the combined effect of suppression technique and working practices aimed at reducing asbestos exposure.

Respirators can reduce the exposure of removal workers to asbestos fibres to 10% or less of the airborne concentrations measured outside the respirator (Williams *et al*, 2007). The majority of participants mainly used the positive pressure mask (68%), resulting in few cases in other categories. Workers who used little or no respiratory protection may still report that they did, diluting differences that may have otherwise been observed. Also important to note is that the effectiveness of any control method is highly dependent on the individual and how closely they follow procedures (Clayton *et al*, 2002).

There was a trend of increasing risk of mortality as the number of hours spent in the stripping enclosure increased. Spending a large number of hours per week in a stripping enclosure exposes workers not only to more asbestos fibres, but also to risk factors associated with working long hours. In particular, high stress because of work demands may increase negative behaviours such as physical inactivity, poor diet, and tobacco and alcohol use (Caruso, 2006). This is in agreement with this study, which found that participants spending long hours in the enclosure were more likely to be current smokers. However, the observed trends remained even after adjustment for smoking habits (not shown).

For all cancers and mesothelioma mortality, the risk decreased as age at first occupational exposure to asbestos increased. Pira *et al* (2005) looked at the mesothelioma death rates in asbestos textile workers using a multiplicative (or relative risk) model. Under this model, age at first exposure was inversely related with mesothelioma risk. Other studies, however, found that age at first exposure had no significant effect on the incidence of mesothelioma (Peto *et al*, 1982; Hansen *et al*, 1998).

With changes in legislation and attitudes towards asbestos usage, the emphasis is now being placed on investigating how effective new regulations and procedures are at reducing the risks associated with occupations in the asbestos industry. However, the length of follow-up since their implementation, in particular the 1983 ALR considered in this study, is still relatively short given the latency periods of asbestos-related diseases. The significant reduction in risk of mortality from all cancers and mesothelioma when the first exposure occurred after the ALR should therefore be treated with caution.

In conclusion, there was no evidence that any particular method of dust suppression during asbestos removal was associated with reduced mortality. A link was suggested between the number of hours spent in the stripping enclosure and smoking status. In addition, the risk of mortality increased as the number of hours spent removing asbestos increased. This study is a first step in assessing the mortality of asbestos removal workers and further follow-up should allow assessment of the efficiency of recent regulations in this respect.

## Figures and Tables

**Table 1:** Characteristics of the asbestos removal workers in the survey and for those included in the analysis (1971–2005)

	**All removal workers**		**Analysed workers**	
	**Number**	**%**	**Number**	**%**
Total	52 387	100.0	31 302	100.0
*Main stripping method*				
Wet	17 634	33.7	17 634	56.3
Dry	9008	17.2	9008	28.8
Both	1413	2.7	1413	4.5
Missing	24 332	46.5	3247	10.4
				
*Main respirator type*				
Positive pressure mask	21 130	40.3	21 130	67.5
Airstream helmet	60	0.1	60	0.2
Full face unpowered mask	5195	9.9	5195	16.6
Half face mask	635	1.2	635	2.0
Minimal	298	0.6	298	1.0
None	80	0.2	80	0.3
Mixed	1443	2.8	1443	4.6
Missing	23 546	45.0	2461	7.9
				
*Average weekly hours spent stripping*				
<10	10 641	20.3	10 641	34.0
10−	3888	7.4	3888	12.4
20−	5333	10.2	5333	17.0
30−	4844	9.3	4844	15.5
40+	4786	9.1	4786	15.3
Missing	22 895	43.7	1810	5.8
				
*Smoking status at last examination*				
Never smokers	13 033	24.9	7924	25.3
Current smokers	29 320	56.0	17 979	57.4
Former smokers	8966	17.1	4889	15.6
Missing	1068	2.0	510	1.6
				
*Age at first exposure (years)*				
<20	10 261	19.6	4642	14.8
20−	21 894	41.8	13 573	43.4
30−	12 262	23.4	8135	26.0
40−	5784	11.0	3626	11.6
50+	2186	4.2	1326	4.2
Missing	0	0.0	0	0.0
				
*Length of time in the survey*				
Short-term	34 165	65.2	17 898	57.2
Long-term	18 222	34.8	13 404	42.8
Missing	0	0.0	0	0.0
				
*Length of exposure (years)*				
<10	37 887	72.3	23 863	76.2
10−	7257	13.9	4096	13.1
20−	4246	8.1	2024	6.5
30−	2164	4.1	926	3.0
40+	833	1.6	393	1.3
Missing	0	0.0	0	0.0
				
*Time of first exposure*				
Pre-ALR	18 055	34.5	5339	17.1
Post-ALR	34 332	65.5	25 963	82.9
Missing	0	0.0	0	0.0

**Table 2:** Standardised mortality ratios (SMR) for all asbestos removal workers in the survey and for those included in the analysis (1971–2005)

	**All removal workers**		**Analysed workers**	
	**Deaths**	**SMR**	**Deaths**	**SMR**
**Cause of death**	**(95% CI)**		**(95% CI)**	
All causes	3165	122.9^**^	985	111.3^**^
	(118.7–127.3)		(104.4–118.4)	
All MN	1274	173.1^**^	384	169.2^**^
	(163.7–182.9)		(152.7–187.0)	
MN of lip, oral cavity and pharynx	19	94.0	6	83.7
	(56.6–146.9)		(30.7–182.2)	
MN of oesophagus	42	88.2	16	104.2
	(63.5–119.2)		(59.6–169.2)	
MN of stomach	49	133.7	18	176.4^*^
	(98.9–176.8)		(104.5–278.8)	
MN of colon	62	127.9	17	119.2
	(98.1–163.9)		(69.4–190.9)	
MN of rectum	43	169.0^**^	15	207.1^*^
	(122.3–227.6)		(115.9–341.5)	
MN of liver (primary)	20	126.6	9	167.8
	(77.3–195.5)		(76.7–318.6)	
MN of larynx	17	212.3^**^	8	322.9^**^
	(123.6–339.8)		(139.4–636.1)	
MN trachea, bronchus & lung	393	200.5^**^	115	215.6^**^
	(181.2–221.4)		(178.0–258.7)	
MN of peritoneum	38	4568.9^**^	12	4367.8^**^
	(3233.2–6271.2)		(2257.0–7629.8)	
MN of pleura	35	1149.3^**^	10	1332.8^**^
	(800.5–1598.4)		(639.1–2451.1)	
Mesothelioma^a^	69	808.2^**^	23	808.2^**^
	(628.8–1022.8)		(512.3–1212.7)	
MN of ovary	0	—	0	—
	(0.0–533.0)		(0.0–7092.9)	
MN of kidney	29	160.1^*^	13	229.0^*^
	(107.2–229.9)		(121.9–391.6)	
MN of bladder	18	94.2	3	61.6
	(55.8–148.8)		(12.7–180.2)	
MN of lymphatic and haematopoietic tissue	80	103.9	24	86.2
	(82.4–129.3)		(55.2–128.2)	
Circulatory disease	981	114.0^**^	258	102.0
	(107.0–121.4)		(89.9–115.2)	
Ischaemic heart disease	665	113.3^**^	171	102.4
	(104.9–122.3)		(87.7–119.0)	
Cerebrovascular disease	147	125.0^*^	41	118.3
	(105.6–146.9)		(84.9–160.5)	
Respiratory disease	222	128.0^**^	44	88.8
	(111.7–146.0)		(64.5–119.2)	
Asbestosis^b^	22	5753.4^**^	3	3849.6^**^
	(3605.6–8710.7)		(794.3–11249.8)	
				

CI=confidence interval.

^*^Significant at *P*⩽0.05; ^**^significant at *P*⩽0.01.

a ICD-10, post-2001.

b Asbestosis determined by underlying cause of death.

**Table 3:** Relative risks (RRs) of mortality for stripping/removal workers, using Poisson regression analyses

	**All causes**			**All MN**			**MN Trachea, bronchus & lung**		
	**Cases**	**RR^a^ (95% CI)**	**LR test (d.f.)**	**Cases**	**RR^a^ (95% CI)**	**LR test (d.f.)**	**Cases**	**RR^a^ (95% CI)**	**LR test (d.f.)**
*Main stripping method*			4.03 (2)			2.35 (2)			2.71 (2)
Wet^c^	397	1.0		141	1.0		48	1.0	
Dry	409	1.1 (0.9–1.2)		169	1.2 (0.9–1.5)		39	0.8 (0.5–1.2)	
Both	48	0.8 (0.6–1.1)		21	1.0 (0.6–1.6)		13	1.3 (0.7–2.7)	
*Main respirator type*			2.82 (6)		10.20 (6)				6.28 (6)
Positive pressure mask^c^	664	1.0		245	1.0		70	1.0	
Airstream helmet	4	—		1	—		1	—	
Full face unpowered mask	127	1.0 (0.8–1.2)		55	1.1 (0.8–1.5)		16	1.1 (0.6–1.8)	
Half face mask	18	0.8 (0.5–1.3)		4	—		1	—	
Minimal	14	0.7 (0.4–1.3)		5	—		2	—	
None	2	—		2	—		1	—	
Mixed	51	1.1 (0.8–1.5)		25	1.4 (0.9–2.2)		11	1.7 (0.9–3.5)	
*Weekly hours spent stripping*			14.97 (4)^**^			1.00 (4)			0.87 (4)
<10^c^	483	1.0		222	1.0		66	1.0	
10−	74	1.0 (0.8–1.2)		32	1.0 (0.7–1.5)		8	0.9 (0.4–1.8)	
20−	125	1.1 (0.9–1.4)		48	1.1 (0.8–1.5)		12	1.0 (0.5–1.8)	
30−	124	1.3^*^ (1.0–1.5)		33	0.9 (0.6–1.3)		9	0.9 (0.4–1.7)	
40+	163	1.4^**^ (1.2–1.7)		44	1.0 (0.7–1.4)		15	1.2 (0.7–2.1)	
*Smoking status*		112.42 (2)^**^				41.07 (2)^**^			67.66 (2)^**^
Never smokers^c^	118	1.0		38			1	1.0	
Current smokers	622	2.5^**^ (2.0–3.0)		216	1.0		86	43.0^**^ (6.0–305.8)	
Former smokers	225	1.5^**^ (1.2–1.9)		125	2.8^**^ (1.9–3.9)		26	16.0^**^ (2.2–118.4)	
*Age at first exposure* (*years)*		14.80 (4)^**^				2.2^**^ (1.5–3.1)			3.22 (4)
<20^c^	184	1.0		91	1.0		21	1.0	
20−	278	1.1 (0.9–1.3)		81	0.9 (0.7–1.2)		18	1.0 (0.5–1.8)	
30−	193	0.9 (0.8–1.1)		70	0.8 (0.6–1.1)		23	1.2 (0.7–2.1)	
40−	211	1.1 (0.9–1.3)		84	0.7^*^ (0.5–1.0)		33	1.2 (0.7–2.0)	
50+	119	0.7^**^ (0.6–0.9)		58	0.6^**^ (0.4–0.8)		20	0.7 (0.4–1.3)	
*Length of time in the survey*			0.01 (1)			14.81 (1)^**^			5.13 (1)^*^
Short-term^c^	419	1.0		116	1.0		33	1.0	
Long-term	599	1.0 (0.9–1.1)		268	1.5^**^ (1.2–1.9)		82	1.6^*^ (1.1–2.4)	
*Length of exposure* (*years)*			16.29 (4)^**^			18.61 (4)^**^			6.88 (4)
<10^c^	553	1.0		166	1.0		49	1.0	
10−	161	1.0 (0.8–1.2)		60	1.1 (0.8–1.5)		26	1.6 (1.0–2.5)	
20−	88	0.8 (0.7–1.0)		45	1.1 (0.8–1.5)		11	0.8 (0.4–1.6)	
30−	94	1.0 (0.8–1.2)		61	1.4^*^ (1.1–1.9)		14	1.0 (0.5–1.8)	
40+	89	1.5^**^ (1.2–2.0)		52	2.1^**^ (1.5–2.9)		15	1.7 (0.9–3.2)	
*Time of first exposure*			0.00 (1)			4.02 (1)^*^			1.43 (1)
Pre-ALR^c^	430	1.0		217		1.0	66	1.0	
Post-ALR	555	1.0 (0.9–1.1)		167		0.8^*^ (0.7–1.0)	49	0.8 (0.5–1.2)	
	**Mesothelioma^b^**			**Circulatory disease**			**Ischaemic heart disease**		
	**Cases**	**RR^a^ (95% CI)**	**LR test (d.f.)**	**Cases**	**RR^a^ (95% CI)**	**LR test (d.f.)**	**Cases**	**RR^a^ (95% CI)**	**LR test (d.f.)**
*Main stripping method*			3.25 (2)			6.81 (2)^*^			8.61 (2)^*^
Wet^c^	12	1.0		109	1.0		77	1.0	
Dry	10	0.9 (0.4–2.1)		107	1.0 (0.7–1.3)		66	0.9 (0.6–1.2)	
Both	0	—		7	0.4^*^ (0.2–0.9)		3	—	
*Main respirator type*			10.14 (6)			1.54 (6)			2.24 (6)
Positive pressure mask^c^	20	1.0		172	1.0		110	1.0	
Airstream helmet	0	—		1	—		1	—	
Full face unpowered mask	1	—		34	1.0 (0.7–1.4)		24	1.1 (0.7–1.7)	
Half face mask	0	—		7	1.1 (0.5–2.5)		5	—	
Minimal	0	—		4	—		2	—	
None	1	—		0	—		0	—	
Mixed	1	—		12	1.0 (0.5–1.7)		9	1.2 (0.6–2.3)	
*Weekly hours spent stripping*			2.29 (1)			12.34 (4)^*^			11.42 (4)^*^
<10^c^	17	1.0		133	1.0		86	1.0	
10−	6	0.5 (0.2–1.3)		16	0.8 (0.5–1.4)		12	1.0 (0.5–1.8)	
20−	—			28	1.1 (0.7–1.6)		16	0.9 (0.6–1.6)	
30−	—			32	1.4 (0.9–2.1)		24	1.6^*^ (1.0–2.6)	
40+	—			47	1.7^**^ (1.2–2.4)		32	1.9^**^ (1.2–2.8)	
*Smoking status*			2.27 (2)			33.41 (2)^**^			35.14 (2)^**^
Never smokers^c^	5	1.0		38	1.0		19	1.0	
Current smokers	7	0.7 (0.2–2.3)		161	2.1^**^ (1.4–2.9)		114	2.9^**^ (1.8–4.7)	
Former smokers	11	1.5 (0.5–4.4)		53	0.9 (0.6–1.4)		34	1.2 (0.7–2.1)	
*Age at first exposure* *(years)*			22.12 (1)^**^			12.38 (4)^*^			12.71 (4)^*^
<20^c^	14	1.0		44	1.0		33	1.0	
20−	9	0.1^**^ (0.1–0.3)		41	0.8 (0.5–1.2)		19	0.5^*^ (0.3–0.9)	
30−	—			51	1.0 (0.7–1.6)		34	0.9 (0.6–1.5)	
40−	—			78	1.5^*^ (1.0–2.2)		54	1.3 (0.9–2.1)	
50+	—			44	0.9 (0.6–1.4)		31	0.9 (0.5–1.5)	
*Length of time in the survey*			8.23 (1)^**^			0.00 (1)			0.60 (1)
Short-term^c^	3	1.0		102	1.0		72	1.0	
Long-term	20	4.5^*^ (1.3–16.0)		156	1.0 (0.8–1.3)		99	0.9 (0.6–1.2)	
*Length of exposure* (*years)*			18.59 (1)^**^			7.19 (4)			10.39 (4)^*^
<10^c^	—			141	1.0		92	1.0	
10−	—			48	1.0 (0.7–1.4)		36	1.2 (0.8–1.7)	
20−	9^d^	1.0		27	0.8 (0.5–1.2)		14	0.6 (0.3–1.0)	
30−	14	7.3^**^ (2.5–21.6)		20	0.6^*^ (0.4–0.9)		13	0.6^*^ (0.3–1.0)	
40+	—			22	0.9 (0.6–1.5)		16	1.1 (0.6–1.9)	
*Time of first exposure*			13.15 (1)^**^			0.46 (1)			0.84 (1)
Pre-ALR^c^	19	1.0		123	1.0		81	1.0	
Post-ALR	4	—		135	1.1 (0.8–1.4)		90	1.2 (0.8–1.6)	
	**Cerebrovascular disease**			**Respiratory disease**					
	**Cases**	**RR^a^ (95% CI)**	**LR test (d.f.)**	**Cases**	**RR^a^ (95% CI)**	**LR test (d.f.)**			
*Main stripping method*			0.62 (2)			2.46 (2)			
Wet^c^	18	1.0		23	1.0				
Dry	14	0.8 (0.4–1.5)		13	0.6 (0.3–1.2)				
Both	2	—		4	—				
*Main respirator type*			7.12 (6)			4.02 (6)			
Positive pressure mask^c^	31	1.0		33	1.0				
Airstream helmet	0	—		0	—				
Full face unpowered mask	4	—		4	—				
Half face mask	0	—		0	—				
Minimal	1	—		1	—				
None	0	—		0	—				
Mixed	0	—		3	—				
*Weekly hours spent stripping*			0.68 (2)			3.46 (3)			
<10^c^	21	1.0		21	1.0				
10−	10	1.4 (0.6–2.9)		8	1.1 (0.5–2.5)				
30−	10	1.2 (0.6–2.7)		7	2.0 (0.8–4.6)				
40+	—			8	1.9 (0.9–4.1)				
*Smoking status*			5.96 (2)			14.18 (2)^**^			
Never smokers^c^	12	1.0		2	1.0				
Current smokers	22	0.9 (0.4–1.8)		30	7.4^**^ (1.8–30.6)				
Former smokers	6	0.3^*^ (0.1–0.9)		11	3.8 (0.8–17.2)				
*Age at first exposure* (*years)*			3.04 (4)			4.35 (3)			
<20^c^	6	1.0		6	1.0				
20−	8	1.1 (0.4–3.2)		12	1.7 (0.7–4.8)				
30−	8	1.5 (0.5–4.7)		12	2.1 (0.8–5.3)				
40−	12	2.2 (0.8–6.6)		14	1.0 (0.4–2.6)				
50+	7	1.3 (0.4–4.4)		—					
*Length of time in the survey*			0.45 (1)			0.46 (1)			
Short-term^c^	15	1.0		20	1.0				
Long-term	26	1.2 (0.7–2.3)		24	0.8 (0.5–1.5)				
*Length of exposure* (*years)*			0.94 (2)			7.14 (3)			
<10^c^	24	1.0		25	1.0				
10−	6	0.8 (0.3–1.9)		6	0.4 (0.2–1.1)				
20−	11	0.7 (0.3–1.5)		—					
30−	—			6	1.1 (0.4–2.7)				
40+	—			7	1.9 (0.8–4.4)				
*Time of first exposure*			1.16 (1)			0.01 (1)			
Pre-ALR^c^	18	1.0		22		1.0			
Post-ALR	23	1.4 (0.7–2.8)		22	1.0 (0.5–1.8)				

^*^significant at *P*⩽0.05; ^**^significant at *P*⩽0.01; LR test=Likelihood ratio test of goodness-of-fit; d.f.=degrees of freedom; CI=confidence interval.

a Relative risk adjusted with Poisson regression for age, calendar period and sex.

b ICD-10, post-2001.

c Reference category.

d Categories: <30, 30+.
